# Acupuncture may play a key role in anti-depression through various mechanisms in depression

**DOI:** 10.1186/s13020-024-00990-2

**Published:** 2024-10-04

**Authors:** Peng Li, Jiangna Zhao, Xiuxiang Wei, Longfei Luo, Yuzhou Chu, Tao Zhang, Anning Zhu, Juntao Yan

**Affiliations:** 1 Yueyang Hospital of Integrated Traditional Chinese and Western Medicine, Shanghai University of Traditional Chinese Medicine, Shanghai, 200437 China; 2 Department of clinical medicine, Xiamen medical college, xiamen, China; 3 Rehabilitation Medicine Department, Shenzhen Hospital of Traditional Chinese and Western Medicine , Shenzhen, China

**Keywords:** Acupuncture, Depression, CUMS, Inflammation, Complementary and alternative therapy

## Abstract

Depression has emerged as a significant global health concern, exerting a profound impact on individuals, as evidenced by its high prevalence and associated suicide rates. Considering its pervasive nature, the absence of optimal treatment modalities remains a challenge. Acupuncture has garnered substantial clinical and experimental validation for its efficacy in addressing diverse forms of depression, including postpartum, post-stroke, and adolescent depression. This article endeavors to elucidate the distinctive attributes and underlying mechanisms of acupuncture in the contemporary treatment of depression. Research has demonstrated that acupuncture exerts diverse physiological effects in animal models of depression, encompassing modulation of the brain, serum, and brain-gut axis. These effects are attributed to various mechanisms, including anti-inflammatory and anti-oxidative actions, promotion of neuronal plasticity, neuroprotection, neurotrophic effects, modulation of neurotransmitters, regulation of endocrine and immune functions, and modulation of cell signal pathways. Currently, the therapeutic mechanism of acupuncture involves the engagement of multiple targets, pathways, and bidirectional regulation. Hence, acupuncture emerges as a promising alternative medical modality, exhibiting substantial research prospects and meriting comprehensive worth further study and dissemination.

## Introduction

Depression is a kind of emotional affective disorder, which is characterized by persistent depression, low mood, a series of symptoms such as inferiority, helplessness and anxiety, and even stupor or repeated hallucinations in severe cases, which may eventually lead to suicide [1]. In addition to the high suicide rate and mortality rate, depression also has the characteristics of high lifetime prevalence and repeated attacks [2, 3]. More than 264 million people of all ages worldwide suffer from depression, according to a new study. The suicide rate caused by major depression is already the number one cause of suicide [4, 5]. Nowadays, depression has become one of the main causes of human health problems around the world, leading to serious disability and economic burden, especially in low-income countries [6]. According to the National Health Commission's survey on the prevalence of mental illness in China, the lifetime prevalence rate of depression among Chinese people is 6.9% [7]. Based on this, it is estimated that the number of people suffering from depression in China will exceed 95 million, and the number of people living with depression in China has exceeded 30 million.

Currently, the mainstay of clinical treatment for depression is the regular administration of antidepressants [8, 9]. Include tricyclic antidepressants (TCA), such as imipraminum, selective serotonin reuptake inhibitors (SSRI), such as fluoxetine, serotonin modulators/agonists (VMS), such as vortexetine, and fast antidepressants N-methyl-D-aspartate receptor(NMDAR) antagonists, such as ketamine [10–13]. However, most of the current antidepressants have a certain degree of adverse effects, dizziness, nausea, and gastrointestinal, central nervous system, autonomic nervous system side effects, which require a certain period of time to respond, and have no therapeutic effect on some patients [14–16]. Many alternative therapies have less side effects and good effects on depression, which has attracted the attention of depressed patients and doctors. For example, the non-invasive neuromodulation technology from rehabilitation discipline, Repetitive Transcranial Magnetic Stimulation (rTMS) has been proved to have a good effect on depression, but its cost is higher than that of ordinary antidepressants [17]. Acupuncture also has antidepressant effect and low cost, but the antidepressant mechanism is still unclear and needs further study.

**Fig. 1:**
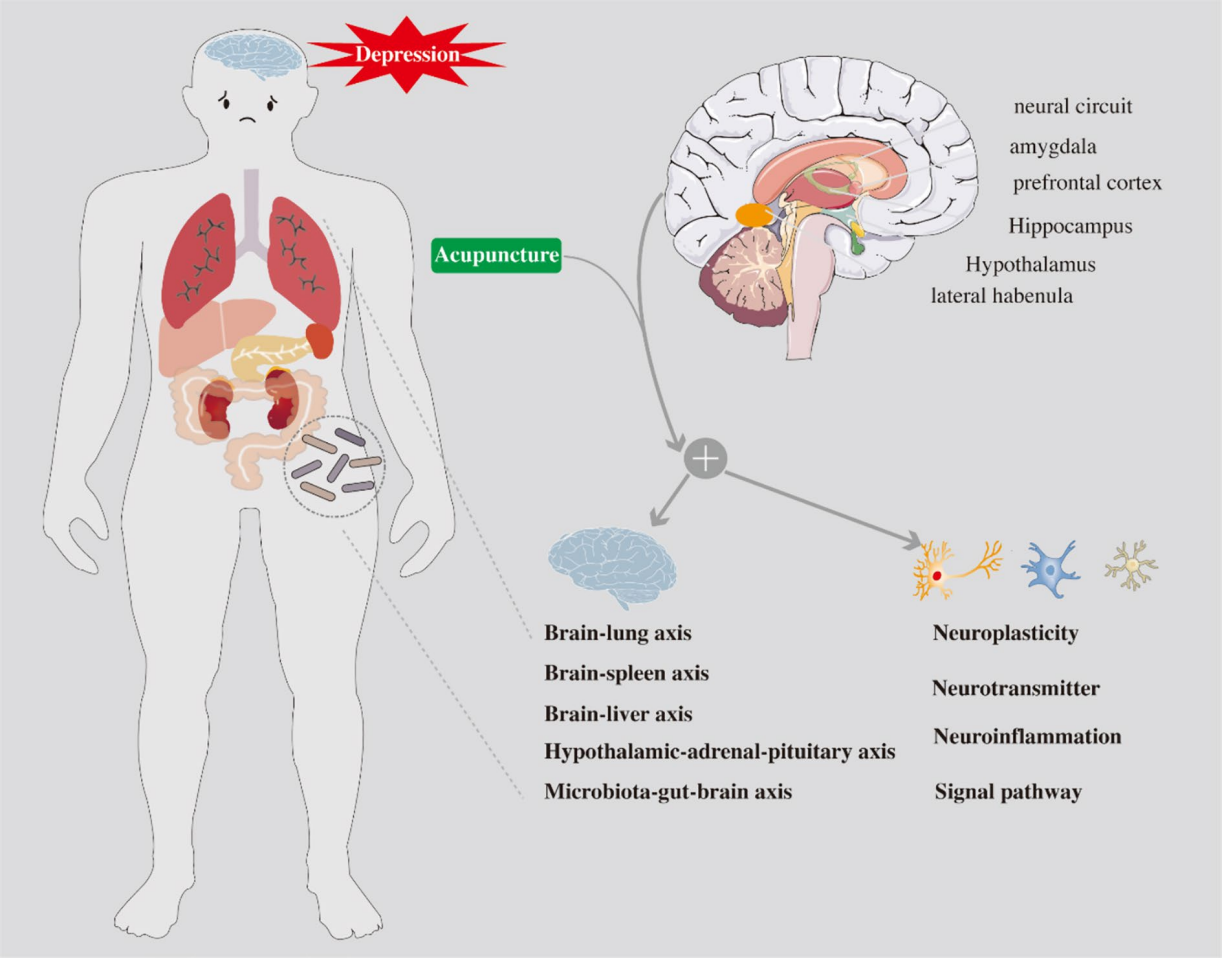
The antidepressant mechanism diagram of acupuncture shows that acupuncture may have therapeutic effect through different brain regions and corresponding mechanisms. Brain regions such as: Hippocampus, amygdala, lateral habenula, prefrontal cortex and neural circuit; Mechanisms such as: Brain-lung axis, Brain-spleen axis, Brain-liver axis, Hypothalamic-adrenal–pituitary axis, Microbiota-gut-brain axis, Neuroplasticity, Neurotransmitter, Neuroinflammation and other Signal pathway

The pathogenesis of depression has not yet been elucidated, and the current mainstream is the monoamine transmitter hypothesis, chronic stress hypothesis, neuroplasticity, inflammation, metabolome, brain-gut, and other hypotheses of depression pathogenesis [18–23]. As shown in Fig. 1. This corresponds to a multitude of depression targets, including the hippocampus (HP), the limbic system neural circuit represented by the amygdala, specific brain regions such as the prefrontal cortex (PFC) and the lateral rein nucleus(LHB), as well as the serum, gut-brain, liver-brain, and splenic-brain axes. The depression model mice are seen to have a smaller hippocampal volume when scanned under Magnetic Resonance Imaging(MRI), and this change can be restored after treatment [24–26]. Activation of neuronal activity of newborn neurons in the hippocampal region does have an inhibitory effect on depression [27]. PFC is interconnected with a variety of neurons, including cognition, and is also involved in many stress-sensitive psychological disorders [28, 29]. Some studies have shown that the biological mechanisms of depression are related to the neuroplasticity of PFC [30, 31]. Depression-like behaviour is mediated by influencing astrocytes which is a key part of PFC and synaptic plasticity [32]. The amygdala contains a variety of neurotransmitters, which are closely associated with depression [33]. Functional magnetic resonance imaging (fMRI) studies suggest that patients with depression have abnormalities in the function of limbic nerve circuits, with a focus on amygdala [34]. LHb can mediate depression through multiple signalling pathways, but mostly through mediating negative aversive signals [35, 36]. In a word, the pathophysiological mechanism of depression is not clear, and the targets involved are also very diverse, which may be a systemic disease [37].

Acupuncture is one of the most important therapies from traditional Chinese medicine, which has been widely used in the treatment of depression. Because of its small side effects, it has been paid more and more attention [38, 39]. Depression is the name of modern medicine, but two thousand years ago, similar depression was recorded in ancient Chinese medicine. It has also been found that women are more prone to this type of illness than men, which aligns with the results of epidemiological studies on depression [40]. More than 400 years ago, Chinese doctors proposed the treatment of depression, in addition to drugs, positive psychotherapy is very important, which is of great significance to the understanding and treatment of depression in traditional Chinese medicine. After more than 2,000 years of development, TCM has formed a unique understanding of the diagnosis and treatment of depression, and has accumulated rich experience [41, 42]. Traditional Chinese medicine believes that depression is a systemic disease, and the internal organs may lead to depression. This is also consistent with the current research in modern medicine. Many effective treatments have been summed up, which are worthy of in-depth study. Chinese medicine treatment of depression also includes acupuncture, massage, music therapy, scraping, cupping and auricular point therapy [43–46].

## Mechanism of acupuncture for depression

Acupuncture is one of the most important treatments for depression in complementary and alternative therapy. Due to its clear therapeutic effect and minimal side effects, it has gradually attracted the attention of researchers all over the world [47]. The general therapeutic principle of acupuncturists in treating depression are regulating the mind, regulating the yin and yang, and harmonizing the qi. Selecting the appropriate acupoints is a key factor in ensuring the effectiveness of acupuncture treatment for depression. The acupoints usually selected in the experimental study of acupuncture and moxibustion for depression mainly include the following, such as Fengfu(GV16), Baihui(GV20), Shangxing(GV23), Shenting(GV24) Renzhong(GV26), Yintang(EX-HN3), Guanyuan (Ren4), Neiguan(PC6), Daling(PC7), Taichong(LR3), Zhongfeng(LR4), Ququan(LR8), Qimen(LR14), Hegu(LI4),Quchi(LI11), Jingqu(LU8), Xinshu(BL15), Ganshu (BL18), Pishu(BL20), Shenshu(BL23), Zhusanli(ST36), Fenglong(ST40), Sanyinjiao(SP6), Yinlingquan(SP9), Yanglingquan (GB34), Yingu(KI10) and so on [48, 49]. Some researchers have also confirmed that abdominal acupuncture can activate the brain's cortical reward circuit to resist depression [50]. However, the mechanism of acupuncture is still unclear, and animal experimental research on acupuncture treatment for depression is of great significance in exploring its mechanism of action [51]. At present, it is known that acupuncture therapy involves many therapeutic effects, acupuncture can regulate central neurotransmitters, inhibit HPA axis hyperactivity, increase BDNF expression, mprove neuroplasticity, inhibitory neuroinflammatory responses, mediated inhibition of the LHb, affect MAPK-related cell signal pathways, affect the epigenetic regulation and regulate the “brain + X axis” [52–55]. Animal models involving depression mainly include chronic unpredictable mild stress(CUMS), chronic restraint stress(CRS), Wistar Kyoto(WKY), maternal separation rats(MS), lipopolysaccharide(LPS), post-stroke depression(PSD), forced swimming (FS), Chronic Social Defeat Stress,(CSDS), Ovariectomy(OVX), Corticosterone(CORT), socially isolated(SI) and so on. We sorted out and summarized the mechanism of acupuncture in treating depression, hoping to provide some references and ideas for revealing the mechanism of acupuncture in treating depression.

### Acupuncture regulates central neurotransmitters

The monoamine hypothesis suggests that depression is related to a deficiency of monoamine neurotransmitters in the nervous system, particularly Norepinephrine (NE) and 5-hydroxytryptamine(5-HT). The use of fluoxetine, a representative 5-HT reuptake inhibitor, has demonstrated certain efficacy in treating depression, providing the most compelling evidence for the monoamine hypothesis and becoming an important target for the development of new antidepressants [56, 57]. However, with the continuous evolution of modern medicine's understanding of depression and the fact that fluoxetine is ineffective for many depressed patients, it is clear that the monoamine hypothesis has significant limitations and can not explain the phenomenon of antidepressants having a delayed onset of action [58, 59].

**Fig. 2:**
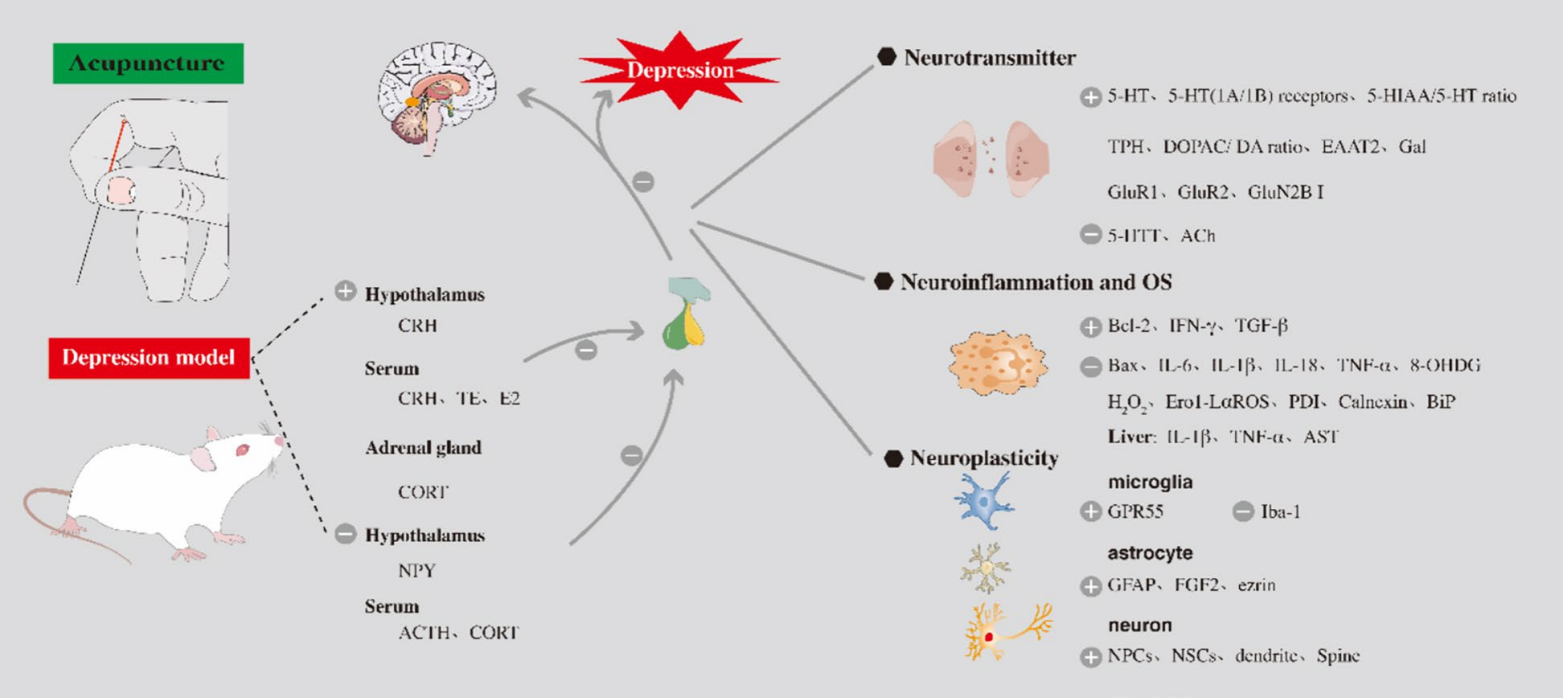
Acupuncture may treat depression-like behavior by influencing neurotransmitters neuroplasticity, neuroinflammation and OS. Such as 5-HT, HPA axis

Numerous studies have found that acupuncture can regulate the release and activity of monoamine neurotransmitters [60, 61]. Evidence has been discovered in multiple animal models showing acupuncture's regulation of 5-HT-related signals, as demonstrated in Fig. 2. In CRS depression mice, research has shown that acupuncture, specifically KI10, LR8, LU8, and LR4, can alleviate depression-like behavior. After acupuncture, treatment, the activation of neurons in the hippocampus, PFC, motor cortex, insular cortex, thalamus, and hypothalamus of depressed mice increased. Additionally, acupuncture elevated the expression of 5-HT1A receptors in the cortex, hippocampus, thalamus, and hypothalamus, as well as 5-HT1B in the cortex and thalamus [62]. In WKY depression model rats, studies found that EA at GV20 and EX-HN3 significantly lowered the expression of 5-HTT protein in the hippocampal CA1 region, exerting an antidepressant effect [63]. In CUMS depression model rats, EA treatment at GV20 and EX-HN3 elevated the expression level of 5-HT1A receptors and alleviated depressive symptoms in the rats [64]. In addition, acupuncture at HT7 relieved depressive-like behavior in mother-infant separated mice and reversed the increased 5-HIAA/5-HT ratio in PFC caused by maternal-infant separation, while also down regulating 5-HTT expression [65]. Acupuncture treatment significantly increased hippocampus 5-HT levels and markedly raised the expression of tryptophan hydroxylase (TPH), 5-HT1A protein, and mRNA in the hippocampus of depressed rats [66]. Acupuncture at HT7 and ST36 reduced the changes in the 3,4-dihydroxyphenylacetic acid (DOPAC) to dopamine (DA) ratio in PFC and HP of MS rat pups, indicating its potential in relieving depressive-like symptoms [67]. Acupuncture improved depressive-like behavior in depression model rats, increased the expression of glial glutamate transporter Recombinant Excitatory Amino Acid Transporter 2(EAAT2) in the hippocampus and PFC, and showed changes in protein and mRNA expression levels [68]. Some studies suggest that EA treatment in CUMS depression model rats may potentially be related to the upregulation of hippocampus galanin (Gal) expression [69]. Acupuncture treatment led to a significant decrease in Acetylcholine (Ach) expression and a substantial increase in acetylcholinesterase (AChE) expression. It increased the expression of glutamate receptor 1 (GluR1), glutamate receptor 2 (GluR2) in the rat brain, and was able to reverse the depressive-like behavior caused by the hypercholinergic state in the depression model [70, 71]. A large number of studies have confirmed the effect of acupuncture on brain neurotransmitters, which is related to the antidepressant effect of acupuncture. However, further evidence on how acupuncture affects neurotransmitters remains to be studied, which may be the research direction of acupuncture antidepressant mechanism.

### Acupuncture inhibits HPA axis hyperactivity

Chronic stress has long been considered one of the important causes of human depression, although it is not a necessary condition [72, 73]. Chronic stress can lead to abnormal gene expression, and this abnormal gene expression can further promote or exacerbate depression [74], Relieving stress can have an anti-depressive effect and enhance neuroplasticity [75]. Another result of this theory is the HPA axis, which is considered to be an important circuit involved in regulating stress response [76]. Research has shown that the HPA axis can affect the secretion of adrenal cortex hormones, affecting corresponding central nervous system areas, with well-studied mechanisms in areas such as the hippocampus and amygdala [77, 78]. Another important piece of evidence is that half of depression patients exhibit HPA axis abnormalities, and this hyperactive abnormality can be alleviated by antidepressants [79, 80]. Furthermore, neurological dysfunction caused by chronic stress also affects the HPA axis. The relationship between chronic stress, the HPA axis, and depression still requires further study [81].

Acupuncture has been shown to regulate the dysregulated HPA axis [82, 83]. As shown in Fig. 2. Research on rats with depression revealed that acupuncture at ST36 and Ren4 points may have an anti-depressive effect by regulating the abnormality of the HPA axis [84]. In a model of HPA axis disruption in rats induced by repeated Corticosterone (CORT) injections, acupuncture at the PC6 point significantly reduced the increase in NPY expression in the hypothalamus, and suppressed the symptoms of low-activation HPA axis in the model of chronic CORT induced depression rats [85]. EA at the auricular concha region was able to lower plasma cortisol and adrenocorticotropic hormone levels induced by CUMS to normal levels by regulating the hyperactivity of the HPA axis and exerting an anti-depressive effect [86]. In male SD rats in the social isolation (SI) depression model, acupuncture embedding at acupoints Du20, EX-HN3, BL23, BL20, BL18, BL15, and Ren4 led to increased testosterone and estradiol levels, and reduced CORT levels. The anti-depressive effect of acupuncture may be related to the regulation of hormone imbalance caused by social isolation [87]. EA at ST36 lowered the content of CORT in the serum of CUMS rats and increased the level of CORT in the adrenal gland, as well as increased the levels of CRH in the hypothalamus and serum, indicating its close association with HPA axis regulation. In addition, EA can also improve rat depressive-like behavior by adjusting glucose levels in the serum, lowering levels of valine and alanine in the serum, thereby regulating glucose metabolism to improve depressive symptoms in rats [88]. Acupuncture can regulate the phenomenon of HPA axis hyperactivity caused by depression, but this may be the manifestation of acupuncture's anti-depression. As for the mechanism of acupuncture's anti-depression, it remains to be discussed.

### Acupuncture improves neuroplasticity

Neuroplasticity is considered to be closely related to depression [89]. The theory of neuroplasticity can well explain the response period of antidepressants, as well as morphological changes such as reduced dendritic spines in neurons in the hippocampus and the phenomenon of decreased hippocampal volume in patients with depression [90]. Clinical data have shown that some depressed patients have significantly reduced levels of BDNF. BDNF is essential for normal physiological function of brain neurons [91], and plays an important role in the growth and differentiation of neurons in the brain [92]. BDNF is a molecule associated with neuroplasticity and is believed to play an important role in regulating neural development [93, 94], and is involved in the pathological and physiological progression of depression [95]. Taking antidepressants can increase BDNF levels in patients and can also enhance the antidepressive ability of animals [96], BDNF can increase the neuroplasticity of relevant brain regions and has become a hot topic in acupuncture antidepression research.

Acupuncture has long been widely demonstrated to improve neuroplasticity [97, 98]. EA can exert an antidepressant effect by increasing the expression of BDNF expression [99, 100]. After EA intervention in the CUMS rat model, hippocampal tissue plasminogen activator (tPA), BDNF, and Tyrosine Kinase receptor B(TrkB) were all be promoted [101]. EA at LI 4 and LR3 acupoints was shown to effectively alleviate depressive state in PSD rats, and its mechanism is possibly related to the up regulation of BDNF/TrkB expression [102]. Furthermore, research has revealed that EA may regulate the interaction of BDNF-TrkB in the midbrain ventral dopaminergic pathway, thus alleviating depressive symptoms [103]. In the FS induced depression mouse model, acupuncture at GV20 and EX-HN3 significantly increased the expression of nerve growth factor (NGF), BDNF, NT-3, and NT-4/5 [104]. Acupuncture can activate BDNF and its downstream signaling pathways, and acupuncture at ST-36 upregulated the expression of BDNF, TrkB, p75 neurotrophin receptor (p75NTR), protein kinase B (Akt), and extracellular signal-regulated kinase (ERK1/2) in the dentate gyrus (DG) in hippocampus [105]. EA at GV20 and SP9 can promote the proliferation of adult neural stem cells (NSCs) and the activation of ERK in the hippocampus of CUS depression model rats, and it has been confirmed in vitro by using EA-treated hippocampal micro dialysis solution of stressed rats [106]. Acupuncture improved the depressive-like behavior of PSD rats, enhanced the activity of the CREB/BDNF/TrkB signal pathway, and promoted phosphorylation of CREB [107]. Signaling pathways are closely linked to the occurrence of depression [108], and acupuncture has been shown to be associated with multiple cellular signaling pathway such as MAPK, including ERK, Jnk and other ways [109, 110]. Acupuncture increases the phosphorylation levels of extracellular signal-regulated kinase (ERK) and cAMP response element-binding protein (CREB) in the hippocampus and PFC, and its antidepressant effects might be mediated through activating the ERK-CREB pathway in the brain [111].

In addition, MEK inhibitors can inhibit the improvement effect of acupuncture on ERK signaling pathway [112]. Another study found that EA at GV20 and EX-HN3 increased the activation of the stress-induced ERK/mitogen-activated protein kinase (MAPK) cascade, with the therapeutic effect of EA being weakened by an ERK inhibitor [113]. Acupuncture at EXHN3 and GV20 hippocampus can also enhance the phosphorylation levels of ERK and P38 to play an antidepressant role [114]. Acupuncture can significantly improve the depression symptoms and adenylate cyclase (AC)/cyclic adenosine monophosphate (cAMP)/dependent protein kinase A(PKA) signaling transduction pathway dysfunction caused by CMS, with EA's antidepressant effects being completely eliminated by a specific PKA inhibitor [115]. Acupuncture can reverse the down regulation of PKA-α and p-CREB in hippocampus caused by CMS [116]. In CUMS depression model rats, acupuncture at GV16 and GV23 adjusts and promotes the recovery of neuroplasticity-related protein kinase Mζ (PKMζ) in PFC and increases the dendrite length and spike density of neurons [117]. Additionally, research indicates that the potential antidepressant effect of acupuncture may be mediated by promoting the proliferation of hippocampal neuronal progenitor cells(NPCs) and precursor cells in CUMS-induced depressive rats [118, 119]. Many studies have found the influence of acupuncture on BDNF, but the research on how acupuncture can increase BDNF is less in-depth, and the direct relationship between acupuncture and BDNF needs to be further discovered and improved.

Research has shown that EA at GV20 and EX-HN3 can reverse the decrease in perineuronal nets (PNN) expression in the PFC caused by the CUMS model, with a confirmation of decreased glutamate decarboxylase 67(GAD67) and Postsynaptic Density-95(PSD95) expression being restored [120]. Another study found that the mechanism of EA in treating rat depression may be related to its up regulation of mammalian target of rapamycinm (TORC1),PSD95, synapsin(SYN), and GluR1 expression, increasing dendritic spine density, and enhancing synaptic plasticity [121]. EA can regulate the levels of glutamate in the hippocampus of CUMS-induced depressive rats, significantly increasing the expression of NR2A in the hippocampus and reducing the expression level of the glutamate ion receptor NR2B [122]. EA treatment of LPS-induced depression mice was found to enhance matrix metalloproteinases (MMPs) expression, subsequently remodeling the neuronal extracellular matrix (ECM), ultimately repairing the neural circuit and enhancing synaptic plasticity to achieve its antidepressant effect [123]. In acupuncture treatment of socially isolated (SI) depressive rats, the phenomenon of reduced neuronal dendrite length and spine density in the CA1 region of the hippocampus was reversed, promoting dendritic remodeling in the hippocampus [124]. EA at LI 4 and LR3 acupoints in CUMS depression rat model enhanced the plasticity of synaptic structures, and upregulated the expression of protein interacting with Cαkinase 1(Pick1) and growth associated protein-43(GAP-43). The AMPA-selective glutamate receptor (AMPAR) antagonist can block the antidepressant effect of EA [125]. Research also found that the mechanism of EA's antidepressant effect may be related to promoting neurogenesis and up regulating the level of transforming growth factor β3 (TGF-β3) protein in the hippocampus [126].

Astrocytes play a significant role in neurogenesis [127], and are closely related to the onset of depression [128]. Research suggests that acupuncture at ST36 and LI11 points can promote astrocytes proliferation to improve neuroplasticity and have an anti-depressive effect [129]. It was observed that acupuncture in the GV20 and EX-HN3 points in CUMS depression rats upregulated the expression of hippocampus basic fibroblast growth factorb (FGF), leading to increased activation of astrocytes to improve depressive-like behavior [130]. Acupuncture at ST36 can reverse the depressive-like behavior, shrinkage of astrocytes in PFC, and decrease in ezrin expression, indicating that astrocytes could be one of the targets of acupuncture treatment of depression [131]. Acupuncture can improve nerve plasticity by improving the expression of nerve cells and glial cells, which may also be the antidepressant mechanism of acupuncture. Follow-up research should pay more attention to the direct influence of acupuncture on astrocytes and microglia.

### Acupuncture in inhibitory neuroinflammatory responses

In recent years, many researchers have proposed the inflammatory theory of depression, as many depression patients exhibit immune dysregulation [132, 133]. Extensive studies have indicated a direct association between inflammation and depression [134, 135]. It has been shown that pro-inflammatory cytokines such as interleukin-1β (IL-1β) and tumor necrosis factor (TNF) can activate systemic inflammatory responses, directly or indirectly mediating the onset of depression [134, 136]. Studies have shown that inflammatory factors such as interleukin-6 (IL-6) and C-reactive protein (CRP) are associated with the occurrence of adult depression [137, 138]. Some studies have shown that Calmodulin-Dependent Kinase II-β (β-CaMKII) in the hippocampal CA1 region can mediate neuroinflammatory responses in depression [139]. Acupuncture at GV20 and GV24 has a regulatory effect on the expression of key proteins in the hippocampal CaMK signaling pathway associated with depression [140]. EA can alleviate the density of dendritic spines and the number of neurons in the hippocampus of CUMS model rats, and its anti-depressive molecular mechanism may involve the GluN2B/CaMKII/CREB signaling pathway [141]. Several hypotheses have been proposed regarding the role of microglia in the pathogenesis of depression, suggesting that microglia play a crucial role in disrupting neural plasticity and exerting harmful effects on neuroprotection, leading to the exacerbation of neuroinflammation and depression [142]. Microglia affect neural plasticity and contribute to a bidirectional neuroimmune pathway in depression [143]. Many molecular pathways mediating depression are interconnected through microglia-related neuroinflammation and hippocampal degeneration [144].

**Fig. 3:**
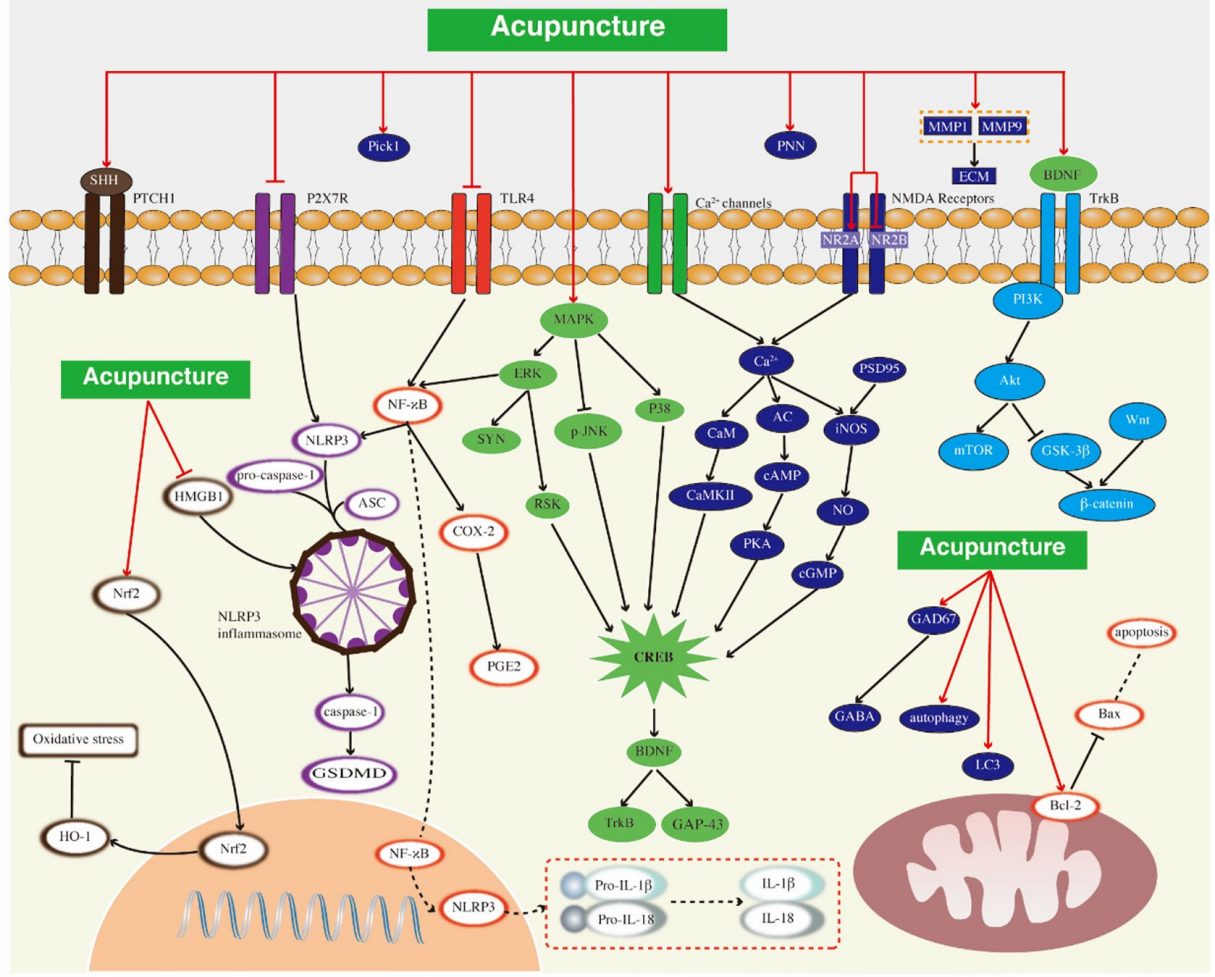
Acupuncture may treat depression-like behavior by affecting different cell signal pathways. Such as JNK, ERK, NLRP3, NMDAR, NO-cGMP, HO-1. The bilayer structure at the bottom of the Figure includes nucleus and mitochondria respectively, and the bilayer structure at the top is cell membrane structure

Acupuncture is considered to have anti-neuroinflammatory effects [145–147]. As shown in Fig. 3. Studies have found that acupuncture at GV20 and EX-HN3 can regulate the levels of cytokines such as IL-1β and IL-6 in the hippocampus and PFC, thereby inhibiting inflammatory responses to alleviate depression [148]. Acupuncture down regulates the expression levels of Bax and caspase-3 and upregulates the expression of B-cell lymphoma-2(Bcl-2) in the CUMS model, and significantly reduces the levels of TNF-α, nuclear factor-κB (NF-κB) and IL-18 in CUMS rats [149]. Acupuncture significantly reduces the levels of NLRP3 inflammasome components and inflammatory cytokines in PFC [150]. Acupuncture has also been found to improving the expression of NLRP3, ASC, caspase-1, Gasdermin-D (GSDMD), and High mobility group box-1 protein(HMGB1) in the serum and hippocampus, restoring microglia, astrocytes and neurons in the hippocampus [151]. EA treatment has a protective effect on LPS-induced depression-like behavior, and its related mechanism may be related to inhibiting inflammation, regulating IDO-mediated tryptophan degradation pathway and inhibiting NR2B activation [152]. In addition, some studies have found that acupuncture may play an anti-depressive role by regulating inflammation and immune response, through base upregulation related to toll-like receptor signaling pathway and NOD-like receptor signaling pathway [153].

Research has shown that acupuncture can prevent CMS-induced depression-like behavior by inhibiting the NF-κB/NLRP3 inflammatory pathway. CMS induces nuclear translocation of NF-κB, increases the levels of p-NF-κB proteins in the hippocampus, and raises the levels of NLRP3, which can be reversed by acupuncture [154]. In the CUMS model, EA at GV 20 and GB 34 play an antidepressant role, which is related to the significant decrease of some inflammatory components of NLRP 3 and the expression of mature IL-1β. Concurrently, EA treatment can reverse the increased expression of P2X7 receptors, ionized calcium binding adaptor molecule 1(Iba-1) and the decreased expression of glial fibrillary acidic protein (GFAP) induced by CUMS, mitigating hippocampal neuroinflammation [155]. The down regulation of G protein-coupled receptor 55(GPR55) in chronic social defeat stress (CSDS) may mediate depressive-like phenotypes, and EA alleviates CSDS-induced depression-like behavior, an effect that is reversed by a GPR55 antagonist. The antidepressant mechanism of EA might be related to the activation of GPR55 in the hippocampus and its anti-inflammatory neuroprotective effects [156].

Studies have found that EA treatment at BL23, GV20, and SP6 improved changes in glycogen synthase kinase-3β (GSK-3β), β-catenin and p-β-catenin induced by CUMS and OVX. This may be achieved by activating the Wnt/β-catenin signaling pathway to promote hippocampal neuronal proliferation in depression model rats [157]. Studies have also found that acupuncture at GV23 and PC7 can also have antidepressant effects by inhibiting the activation of NO-cGMP signaling pathway in hippocampus caused by CUMS [158]. EA at GV20 and EX-HN3 can regulate the expression levels of Prkc and Mapt, indicating that EA can alleviate depression by regulating the MAPK signaling pathway [159]. EA treatment reduced the expression of c-JUN and c-JUN terminal kinase (JNK) in the hippocampus of CUMS depression model rats, suggesting that EA alleviates depression by regulating the MAPK/JNK signaling pathway [160]. Acupuncture has been found to have a wide range of anti-inflammatory effects, specifically, it can reduce pro-inflammatory factors such as IL-1β and increase anti-inflammatory factors such as IL-10. The anti-inflammatory effect of acupuncture and moxibustion is closely related to the anti-depression mechanism, which has been proved by a large number of animal experiments. However, how acupuncture and moxibustion can play an anti-inflammatory role and achieve anti-depression effect still needs further study, which also suggests the research direction of anti-inflammatory mechanism of acupuncture.

### Acupuncture-mediated inhibition of the LHb

LHb, which due to the specificity of its anatomical location, can be projected to multiple nuclei and nerve fibres [161]. LHb plays an increasingly important role in neuroscience, particularly in depression [162]. In 2013, Hu's group found that β-camKII of the lateral habenula nucleus plays a key role in mediating depression [163]. And in 2018, it was revealed that the mechanism of ketamine's rapid antidepressant action is still to prevent the abnormal cluster discharge of the lateral habenula nucleus, and a new target of depression in the lateral habenula nucleus, Inwardly rectifying potassium channel subtype 4.1 (Kir4.1) [164, 165]. AP improves the area percentages of IBA-1, GFAP, BrdU, and DCX in the LHb of CUMS rats and increases the expression of BDNF/TrkB/CREB, suggesting a potential antidepressant mechanism of acupuncture [166]. The lateral habenular nucleus is a frontier target for mediating depression, and the effect of acupuncture on the lateral habenular nucleus deserves further study.

### Acupuncture improves oxidative stress and mitochondrial autophagy

Oxidative stress (OS) and mitochondrial autophagy play crucial roles in various diseases. Depression patients commonly experience mitochondrial dysfunction. Mitochondrial abnormalities may lead to disrupted energy metabolism, contributing to depression. Abnormal mitochondrial function may also lead to OS, inflammation, and apoptosis, all closely associated with the onset and development of depression [167], Mitochondria are also involved in the synthesis and regulation of neurotransmitters and involved in major neurological and psychiatric diseases [168]. Acupuncture has been found to alleviate OS and mitochondrial autophagy [169, 170].

Studies have found that acupuncture can alleviate depressive-like behaviors induced by CUMS in rats, and in PFC and hippocampus, CUMS-induced increases in NO, PGE2, iNOS, and COX-2 were significantly reduced after acupuncture [171]. Acupuncture also significantly reduces OS products such as ROS and H_2_O_2_, and up regulates the expression of nuclear factor-erythroid 2-related factor 2,(Nrf2) and heme oxygenase-1(HO-1), exerting its antidepressant effect by regulating Nrf2/HO-1 to reduce OS products [172]. EA at ST36 and GV20 relieves neuronal damage in LPS-exposed mice, inhibiting OS, improving mitochondrial respiratory function, energy metabolism, and mitochondrial morphology, and improving LPS-induced hippocampal damage through the HO-1-mediated modulation of mitochondrial function [173]. Acupuncture at SP6 showed significant antidepressant-like effects on ovariectomy OVX-induced depression rats, which can reduce endoplasmic reticulum stress and OS in the amygdala, and reduce the expression of related proteins such as 8-OHDG, BiP, p-JNK, PDI, Ero1-Ia, and Calnexin [174]. Acupuncture in a PSD rat model significantly increases GSH levels, and slightly reduces malondialdehyde (MDA), changes that are inhibited by a Shh inhibitor. Acupuncture alleviating depression-like symptoms in PSD rats through the activation of the Shh signaling pathway to suppress inflammation and OS [175]. In experiments, EA in CUMS-induced depression-like rats partially inhibits autophagy by reducing the number of hippocampal autolysosomes and the level of LC3, resulting in its antidepressant effect [176]. EA at GV20 and EX-HN3 regulates mitochondrial proteins, consistent with the results of the ultrastructure study of hippocampal neurons in CUMS rats, confirming that EA may improve depressive behavior in rats by protecting mitochondrial function [177]. Acupuncture reduces the level of oxidative stress-related factors, improves mitochondrial function and affects mitochondrial autophagy in depression model animals, which may be related to its antidepressant mechanism, and its internal relationship deserves further study.

### Acupuncture affects the epigenetic regulation

Many circRNAs are highly expressed in the central nervous system, involved in various regulatory processes, and involved in the pathophysiology of many diseases [178, 179]. However, the potential role of circRNAs in brain diseases, especially major depressive disorder (MDD), is still little known [180]. The role of circDYM and its coupling mechanism in depression has been found, providing transformative evidence that circDYM may be a new therapeutic target for depression [181]. Analysis of gene chip technology found that EA can relieve depression by regulating the expression of specific genes [182], and other studies found that EA can affect miRNA [183].

EA at GV20 and EX-HN3 can improve adult depression-like behavior induced by maternal separation (MS). Genome-wide RNA sequencing was used to study the changes in PFC transcriptome, and the differentiated lncRNA/circRNA-miRNA-mRNA interaction network was analyzed using the principle of competitive competing endogenous RNA (ceRNA). The results showed that EA improved depression-related symptoms by regulating the expression of multiple genes [184]. Other studies found that the antidepressant effect of EA at GV20 and EX-HN3 in CUMS rats may be related to the inhibition of miRNA-16 expression in the hippocampus and serum [185]. The CUMS model down regulates serum and hippocampal BDNF, and down regulates hippocampal acH3K9 and up regulates hippocampal HDAC2 expression. Acupuncture can reverse these changes, and its antidepressant effect may be mediated through the regulation of DNA methylation and histone modification of BDNF [186]. Many researches pay attention to the effect of acupuncture on epigenetics, which is a powerful scientific basis for the therapeutic effect of acupuncture. Epigenetics plays an important role in the occurrence and development of depression, and the influence of acupuncture on epigenetics deserves further study.

### Acupuncture regulates the “brain + X axis”

Depression is a holistic disease, closely related to the whole body. Known studies have found that the pathogenesis of depression is closely related to the microbial-intestinal-brain axis [187–189], brain-spleen axis [190], brain-lung axis [191, 192], and brain-liver axis [193–195]. Homeostasis of intestinal microbes is closely related to the health of the body [196], especially depression [197].

Acupuncture, as a multi-target therapy, is believed to regulate the brain-gut signaling axis [198, 199]. Acupuncture regulates the intestinal microbial disorder caused by CUMS and reduces the relative abundance ratio of Bacteroidetes/Firmicutes in rats [200]. EA can down regulate the expression of DRG Pirt and colon TRPV1 in CUMS rats, regulating the intestinal homeostasis of rats [201], It can also effectively alleviate visceral hypersensitivity in model rats, and may be effective by inhibiting BDNF/TrkB/PLC/Ca^2+^ signaling pathway [202]. In the CUMS combined with acute restraint stress rat model, the contents of 5-HT and calcitonin gene-related peptide (CGRP) in the distal colon, spinal cord, and hypothalamus increased, while neuropeptide Y (NPY) decreased, and EA reversed this change, restoring the brain-gut axis to a balanced level [203]. Studies have found that acupuncture can prevent and reduce CUMS-induced depression-like phenotype, thereby improving inflammation in serum, liver, and intestinal microbiota. Acupuncture reduces depression-like behavior caused by chronic unpredictable mild stress through the gut-liver-brain axis [204]. Studies have found that acupuncture CRS mice reduced the expression of IL-1β and TNF-α in the liver, and changed liver lipid metabolism by weakening leptin insensitivity, reducing aspartate aminotransferase (AST) levels, which is evidence that acupuncture may affect the liver-brain axis to fight depression [205]. Compared with the EA treatment group and the CUMS group, the liver cells and microglia in the EA group had regular morphology and orderly arrangement. EA downregulated the expression of P2X7R/NLRP3/IL-1β and relieved depression-like behavior. P2X7R may be the target of EA intervention in the liver-brain axis to treat depression [206]. Acupuncture may improve depression-like symptoms caused by CUMS by regulating the brain-spleen axis mediated by HMGB1/TLR4 signaling pathway, and reduce the excessive activation of amygdala and peripheral blood inflammation [207]. More and more researches pay attention to the influence of acupuncture on multiple targets of the whole body, such as the brain-intestine axis and brain-spleen axis, which is more in line with the traditional Chinese medicine acupuncture treatment concept of depression, and it is not effective through a single target, which may be closer to the truth of acupuncture anti-depression mechanism.

### Acupuncture regulates the "brain functional network"

In recent years, the research on the antidepressant mechanism of acupuncture and moxibustion using Neuroimaging has gradually attracted scholars' attention [208, 209].It is gradually proved that acupuncture can regulate the functional network of the brain. The clinical study of fMRI brain imaging shows that verum acupuncture plus fluoxetine can significantly increase the rsFC of amygdala and achieve therapeutic effect [210]. There are also other clinical research results suggesting that acupuncture plus fluoxetine may achieve antidepressant effect by regulating rsFC of cortical striatum reward circuit [50]. Using rs-fMRI techonogy combined with functional communication (FC) data processing method, it was found that acupuncture GV20 in patients with depression can affect the functional communication of amygdala, mainly increasing the FC in amygdala with DLPFC, and reducing the functional communication with PAG and insula [211]. Some studies have also found that the persistent effect of electroacupuncture inhibits the overactivation of DMN in patients with depression. The persistent effect is stronger and more extensive than the immediate effect, and triggers brain reaction, leading to the integration of brain networks in patients with depression [212]. With the development of neuroimaging technology, the immediate effect of acupuncture on depression is more likely to be revealed.

## Conclusions

In summary, acupuncture therapy has the characteristics of multi-target and multi-pathway, and can play an important role in the treatment of depression. It should be noted that acupuncture is a complementary or alternative therapy and cannot replace the main treatment. Acupuncture should be performed under the guidance of a professional doctor.

The mechanism of acupuncture treatment for depression, from the perspective of research targets, mainly includes the limbic system nerve circuit represented by the hippocampus and amygdala, as well as specific brain regions such as PFC and lateral striatal nucleus. Serum, Brain-gut axis, brain-liver axis, etc., covering all relevant targets of depression research. The corresponding mechanisms include anti-inflammation, antioxidant, enhancing neuronal plasticity, neuroprotection, neuronutrition, neurotransmitter, immune and cell signaling pathways.

The research on the regulation of central nervous system neurotransmitters by acupuncture was the earliest to be conducted, which paved the way for the animal experimental studies on the antidepressant effects of acupuncture. However, this may only reflect the outcome of acupuncture therapy rather than its underlying mechanism. Acupuncture has been shown to inhibit the excessive activity of the HPA axis, and the regulation of the HPA axis by acupuncture has also been extensively studied and further explored. Acupuncture has been shown to increase the expression of BDNF, and numerous experiments have confirmed this, making BDNF an objective evaluation index for the antidepressant effects of acupuncture. Acupuncture improves neuroplasticity, and the neuroplasticity of depressed animals has been reversibly restored to varying degrees. Acupuncture inhibits neuroinflammation, Acupuncture mediates the suppression of LHb, and the direct or indirect action of acupuncture on LHB is worth further research. Acupuncture affects the MAPK-related cell signaling pathway, and MAPK is closely related to important physiological processes such as cell growth, proliferation, differentiation, and apoptosis, reflecting the effects of acupuncture. Acupuncture affects epigenetic regulation and affects the expression of factors such as BDNF, which is consistent with the research on increasing BDNF and forms a before-and-after corroboration. The research on the regulation of "brain + X axis" by acupuncture is consistent with the concept of acupuncture being a multi-target therapy that can regulate the overall state of the body, and may be closer to the mechanism of action of acupuncture in treating depression.

Experimental studies on acupuncture treatment for depression cover a variety of animal models of depression, but are still mainly based on common model animals such as rats and mice. Primate experimental animals that are more similar to the pathogenesis of human depression have not been involved yet. And high-quality experimental studies on acupuncture treatment for depression are still relatively scarce. The specific mechanism is still unclear and needs further exploration.

## Data Availability

The data used in the article can be obtained from the author of the article through formal channels.

## Abbreviations

TCA: Tricyclic antidepressant
SSRI: Selective serotonin re-uptake inhibitor
SMS: Serotonin modulator and stimulator
NMDAR: N-methyl-D-aspartate receptor
HP: Hippocampus
PFC: Prefrontal cortex
MRI: Magnetic resonance imaging
NO: Nitric oxide
fMRI: Functional magnetic resonance imaging
TCM: Traditional Chinese medicine
HPA: Hypothalamic–pituitary–adrenal
BDNF: Brain-derived neurotrophic factor
CUMS: Chronic unpredictable mild stress
CRS: Chronic restraint stress
WKY: Wistar Kyoto
MS: Maternal separation rats
LPS: Lipopolysaccharide
PSD: Post-stroke depression
FS: Forced swimming
CSDS: Chronic social defeat stress
OVX: Ovariectomy
SI: Socially isolated
CORT: Corticosterone
NE: Norepinephrine
5-HT: 5-Hydroxytryptamine
DOPAC: 3,4-Dihydroxyphenylacetic acid
DA: Dopamine
EAAT2: Recombinant excitatory amino acid Transporter 2
Gal: Galanin
Ach: Acetylcholine
AChE: Acetylcholinesterase
GluR1: Glutamate receptor 1
GluR2: Glutamate receptor 2
tPA: Tissue plasminogen activator
TrkB: Tyrosine Kinase receptor B
NGF: Nerve growth factor
p75NTR: P75 neurotrophin receptor
Akt: Protein kinase B
ERK: Extracellular signal-regulated kinase
DG: Dentate gyrus
NSCs: Neural stem cells
PKMζ: Protein kinase Mζ
GAD67: Glutamate decarboxylase 67
PSD95: Postsynaptic density-95
mTORC1: Mammalian target of rapamycinm
SYN: Synapsin
MMPs: Matrix metalloproteinases
ECM: Neuronal extracellular matrix
SI: Socially isolated
Pick1: Protein interacting with Cαkinase 1
GAP-43: Growth associated protein-43
AMPAR: AMPA-selective glutamate receptor
TGF-β: Transforming growth factor β
FGF: Basic fibroblast growth factorb
IL-1β: Interleukin-1β
TNF: Tumor necrosis factor
IL-6: Interleukin-6
CRP: C-reactive protein
β-CaMKII: Calmodulin-dependent kinase II-β
COX-2: Cyclooxygenase-2
Bcl-2: B-cell lymphoma-2
NF-κB: Nuclear factor-κB
TUNEL: Terminal deoxynucleotidyl transferase-mediated deoxyuridine triphosphate-biotin nick end-labeling
GSDMD: Gasdermin-D
HMGB1: High mobility group box-1 protein
Iba-1: Ionized calcium binding adaptor molecule 1
GFAP: Glial fibrillary acidic protein
GPR55: G protein-coupled receptor 55
EA: Electroacupuncture
Kir4.1: Subtype 4.1
Nrf2: Nuclear factor-erythroid 2-related factor 2
HO-1: Heme oxygenase-1
MDA: Malondialdehyde
MAPK: Mitogen-activated protein kinase
AC: Adenylate cyclase
cAMP: Cyclic adenosine monophosphate
PKA: CAMP-dependent protein kinase A
GSK-3β: Glycogen synthase kinase-3β
JNK: C-JUN terminal kinase
ceRNA: Competing endogenous RNA
CGRP: Calcitonin gene-related peptide
NPY: Neuropeptide Y
AST: Aspartate aminotransferase
